# Can non-carious cervical lesions depth affect clinical response in pain intensity and remaining dentin thickness?

**DOI:** 10.1590/0103-6440202204789

**Published:** 2022-10-21

**Authors:** Alexia da Mata Galvão, Ramon Corrêa de Queiroz Gonzaga, Maria Antonieta Veloso Carvalho de Oliveira, Alexandre Coelho Machado, Gabriella Lopes de Rezende Barbosa, Paulo Vinicius Soares, Gisele Rodrigues da Silva

**Affiliations:** 1 Department of Operative Dentistry and Dental Materials, School of Dentistry, Federal University of Uberlandia, Minas Gerais, Brazil.; 2 Department of Endodontics, School of Dentistry, Federal University of Uberlandia, Minas Gerais, Brazil.; 3 Department of Oral Diagnosis, School of Dentistry,FederalUniversity of Uberlandia; Av. Pará, 1720, Umuarama, Uberlândia, Minas Gerais, Brazil.

**Keywords:** Clinical Competence, Cone Beam Computed Tomography, Dental Pulp Test, Dentin Sensitivity

## Abstract

Non-carious Cervical Lesions (NCCL) are dental tissue defects, non-related to caries, frequently observed in the dental practice. The aim of this study was to evaluate the effects of NCCL on dentin depth and thickness and the response to dental pain by means of clinical diagnostic tests. 86 teeth from 14 patients with NCCL were assessed by: depth of NCCL, clinical tests (evaporative stimulus, to detect pain levels of dentin hypersensitivity, cold thermal test to classify pulp health, percussive stimuli to evaluate the periradicular tissues and cone beam computed tomography (tomography to evaluate remaining dentin thickness (RDT). In terms of depth, the sample was divided into two groups: G1- teeth with NCCLs ≤1.0mm and G2- teeth with NCCLs between 1.1-2.0 mm. Dental pain data were compared by Mann-Whitney test and RDT by Student’s t-test and correlations by the Pearson test (p<0.05). The depth of NCCL does not influence dental pain response to evaporative stimulus (p=0.129), cold thermal test (p = 0.125), vertical (p = 0.317) and horizontal (p = 0.119) percussion clinical diagnostic tests. However, G1 showed more RDT (p<0.001), and the correlation test showed that deeper NCCL presents smaller remaining dentin thickness (p=0.011/r=-0.273). In conclusion, tooth with NCCL up to 2mm-depth presents similar levels of pain for dentin hypersensitivity, pulp and periradicular tissue independent to NCCL depth, however, lesions with ≤1.0mm-depth showed greater RDT in tomographic findings.

## Resumo

Lesões Cervicais Não Cariosas (LCNC) são defeitos do tecido dentário, não relacionados à cárie, frequentemente observados na prática odontológica. O objetivo do estudo foi avaliar os efeitos da profundidade e espessura da dentina de LCNC na resposta à dor dentária por meio de testes de diagnóstico clínico. 86 dentes de 14 pacientes com LCNC foram avaliados por: profundidade da LCNC, testes clínicos (estímulo evaporativo, para detectar níveis de dor de hipersensibilidade dentinária, teste térmico frio para classificar a saúde pulpar, estímulos percussivos para avaliação do tecido perirradicular e tomografia computadorizada de feixe cônico (TCFC), para avaliação da espessura de dentina remanescente (EDR). Em termos de profundidade, a amostra foi dividida em dois grupos: G1- dentes com LCNCs ≤1,0mm e G2- dentes com LCNCs entre 1,1-2,0 mm. Os dados de dor dentária foram comparados pelo teste de Mann-Whitney e a EDR pelo teste t-Student e correlações pelo teste de Pearson (p <0,05). A profundidade da LCNC não influencia a resposta da dor dentária ao estímulo evaporativo (p = 0,129, teste térmico frio (p = 0,125), teste diagnóstico clínico de percussão vertical (p = 0,317) e horizontal (p = 0,119), porém o G1 apresentou maior EDR (p <0,001) e o teste de correlação mostrou que LCNC mais profunda apresenta menor EDR (p = 0,011 / r = -0,273). Conclusão: Dentes com LCNC de até 2mm de profundidade apresenta níveis semelhantes de dor para hipersensibilidade dentinária, pulpar e saúde perirradicular, independente da profundidade da LCNC. Entretanto, lesões com profundidade ≤1,0mmin apresentaram mais EDR nos achados tomográficos.

## Introduction

Tooth structure loss at the cementum-enamel junction that is not associated to the presence of caries has been identified as non-carious cervical lesions and has up to 46.7 % prevalence rate in adults ^1^ ^,^ ^2^. Clinically, non-carious cervical lesions present in different geometries, ranging from mild depressions to large wedges or rounded shapes ^3^. Current studies suggest that the formation and/or progression of these lesions have multifactorial etiology. The association between factors such as corrosion, friction, attrition, and abrasion, besides occlusal stress ^4^ ^,^ ^5^ ^,^ ^6^are involved in cervical dentin exposure and are a predisposing factor of hypersensitivity ^7^. Dentin hypersensitivity can be defined as a short sharp pain that arises from the exposed dentin in response to thermal, tactile, osmotic, chemical, or evaporative stimuli ^8^.

Currently, there are several clinical tests for dentin hypersensitivity such as an air blast, ice stick or mechanical stimulation with an exploratory probe ^9^. One challenge when using these tests in clinical settings is the subjectivity of individual perceptions that affect the values generated by the visual analogue scale ^10^. Dentin hypersensitivity is diagnosed by exclusion and in some cases; it can be misinterpreted as pain caused by pulpitis. Thus, differential diagnosis: pulp sensibility and sensitivity to percussion test are essential to identify the etiopathogenesis of cervical lesions and exclude pulp and apical commitment ^11^.

Many studies have suggested that periapical intraoral radiographs only present loss of structure when it achieves levels of 30 to 40% ^12^ ^,^ ^13^. The detection of periapical lesions in radiographs are affected by tissue densities, X-ray angulation, location and shape of the structures, its inherent two-dimensional projection of the lesions, not exposing their full extent and depth, which may lead to treatment failure ^13^. Cone beam computed tomography could be a frequent imaging technology to analyze the periradicular region in three dimensions in order to overcome those limitations, without overlaps and with better image quality ^13^ ^,^ ^14^.

Still, observational studies that associate depth and pulp sensitivity are not common. Therefore, the aim of this study was to evaluate the effects of NCCL dentin depth and thickness on the response to dental pain when clinical diagnostic tests were applied. The null hypotheses were that: 1) Non-carious cervical lesions depth does not influence dental pain response to evaporative stimulus, cold thermal test, vertical and horizontal percussion tests, and2) The depth of non-carious cervical lesions does not influence the remaining dentin thickness (RDT) by means of cone beam computed tomography.

## Materials and methods

The Institutional Review Board of Federal University of Uberlândia (#1.301.696) approved this observational study. Registry (no. 11968), the individuals included in the study were patients in a rehabilitation program for subjects with NCCL at the School of Dentistry of the Federal University of Uberlândia and all participants signed an informed consent.

The total number of teeth included in the study was 86 (5 incisors, 9 canines, 55 premolars and 17 molars) from 14 individuals (6 to 7 teeth per subject). A statistical test was performed to make sure that the variability of the type of tooth would not influence the outcome of the study. The age ranged from 25 to 50 (means of 48) years old and most patients were older, divided among men and women. Participants included in this study, should be at least 18 years old, in good general and oral health and were required to have non-carious cervical lesions. Teeth with presence of dental caries and restorations or fractures were excluded. Participants who underwent recent periodontal surgery or desensitizing treatment in the last three months and those with dental prostheses and orthodontics apparatus, teeth with or under endodontic treatment. In addition, pregnant and lactating women, individuals with bruxism or any systemic/psychological diseases, anti-inflammatory or analgesic drug users, smokers or patients undergoing tooth-whitening procedures were also excluded. For all individuals, we performed an analysis between 6 and 7 teeth for patient.

### Sample size

To measure sample size calculation, a pilot study was made, tomography scans of patients with non-carious cervical lesions was carried out. With these data, a t-test on Sigma Plot (version 12.0; Systat Software Inc, Chicago, IL, USA) was performed considering a difference between the means (0.290) and standard deviation (0.450), power of the test of 80%, and alpha error of 0.005, totaling a sample calculation of 39 teeth. Thus, these data were not included in our definitive study; 86 teeth was reached due to the need of achieving a minimum of 39 teeth per group, which is dependent on the number of patients.

### Clinical assessments

**Figure 1:**
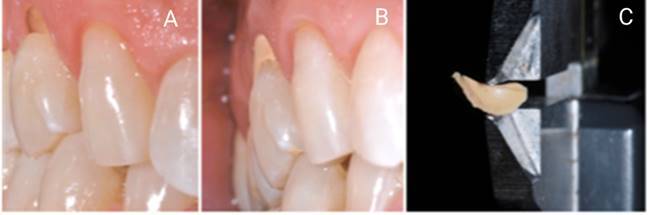
A- Non-carious cervical lesions identification; B- Addition silicone lesion molding, resuming the lost tooth structure; C- Measured with a caliper to identify the depth of the lost tooth structure (NCCL)

Depths of lesions in this *in vivo* study were impressed with polyvinyl siloxane (PVS) elastomeric material (HydroXtreme; Coltene/Whaledent, Altstätten, Switzerland) that was inserted in the lesion to cast the lost structure (Figure 1). This was an initial measurement of the lesion following the methodology of previous studies ^4^ ^,^ ^15^. Because in the first contact with the patient, there was still no CT scan**.** After polymerization, the cast was removed and the depth of the lesion was measured using a digital caliper (Mitutoyo 500-171-30B; Mitutoyo, Santo Amaro, Brazil) ^4^. According to the depth recorded, the sample (n = 86) was divided in two groups: Group 1 (G1) - teeth with NCCLs ≤1.0mm (n=41); Group 2 (G2) -teeth with NCCLs between 1.1-2.0mm (n=45). The cervical region surface was observed macroscopically, all non-carious cervical lesions recognized were counted. To classify the depth, three previously calibrated evaluators visually analyzed the cast obtained in the previous assessment ^1^ ^,^ ^16^. First, tests were performed on healthy teeth. The calibration was carried out using a dental mannequin (training, adjustments and calibration exercises). Then the operator classified lesions under the supervision of an experienced clinician. The calibrated operator's questions were presented to an experienced operator. Only after the calibration, the operator could classify the depth. However, the allocation of teeth in the groups was confirmed by measurements performed later by tomographic images. The depth of each lesion was measured considering the distance (mm) from a vertical line drawn parallel to the cavo-superficial angle and the deepest portion of the lesion.

Each tooth was subjected to an evaporative stimulus to detect pain levels of dentin hypersensitivity, cold thermal test to classify pulp health and, vertical and horizontal percussion to detect periradicular tissues heath. An expert examiner, other than the operator, conducted these tests. First, tests were performed on healthy teeth. The calibration was carried out using a dental mannequin (training, adjustments and calibration exercises). Then, the operator performed the tests under the supervision of the experienced clinician. The calibrated operator's questions were presented to an experienced operator. Only after the calibration, the operator could perform the tests.

An evaporative stimulus (controlled air blast) generated by an air-water syringe was used to determine the tooth sensitivity level. The air jet was perpendicularly directed to the cervical buccal surface of the hypersensitive tooth for two seconds at an approximately 1 cm-distance. The adjacent teeth were protected with a polyester strip to avoid false-positive results. The operator requested the participants to rate their pain according to a 10-point visual analog scale (VAS) and the value was recorded. The recorded values were distributed according to their level: 0 - no pain; 2 - mild pain ^17^; 3 - moderate pain ^6^; 4 - severe pain ^7^ (Gnatus Medical-Dental Equipments, Ribeirão Preto, Brazil).

Cold thermal test was made to test pulp health, with refrigerant spray (Endo-Frost; Coltene/Whaledent, Altstätten, Switzerland) that was applied to a small cotton pellet (SS Plus, Maringá, Brazil) and placed on the middle third of the buccal surface (SSWhite Duflex, Rio de Janeiro, Brazil), for a maximum of 5 seconds or until the participant raised a hand to indicate that he or she felt a cold sensation. Then, pain level was classified as negative, positive or severe.

To test periradicular pain, vertical and horizontal percussion tests were performed. The occlusal surface of the tooth was tapped with mild force using a dental mirror handle (SSWhite Duflex, Rio de Janeiro, Brazil). The test was conducted vertically, then laterally on the incisal edges of anterior teeth and on the buccal and lingual cusps of posterior teeth ^18^.

### Tomographic assessments

Cone beam computed tomography images were acquired in a Gendex CB-500 unit (Gendex Dental Systems, Hatfield, PA, USA) using the following parameters: 120 kV, 5 mA, 14x8 cm FOV and 0.2 mm voxel size. The images were randomly evaluated in blocks of 10 images, using CS 3D Imaging software (version 7; Carestream Health, Rochester, NY, USA), in a secluded, dimly lit room. In relation to the tomographic appraisal, although cone beam computed tomography allows examination of entire structures and provides precise dimensions, shapes and locations, when it comes to discrete conditions, its spatial resolution limits the accuracy in the range of half a millimeter at best ^29^, in addition to its high cost and radiation exposure that are limiting factors for its indication for this specific use. In this sense, cervical lesions are often observed in tomographic images acquired for different or supplementary purposes that often use 0.2mm voxel sizes, as well as the present study. High resolution cone beam computed tomography scans, using smaller voxel sizes, could provide better images for such small lesions, but it would not represent the a common scenario in dental offices. Further studies comparing the influence of this parameter in lesions examination should be performed ^19^.

**Figure 2:**
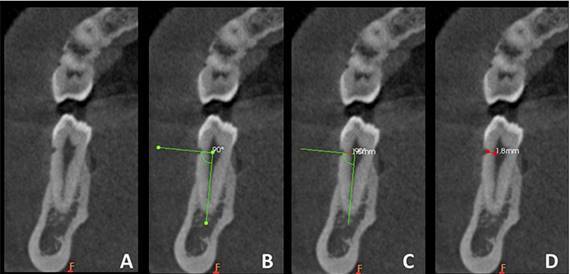
Remaining dentin thickness measurement: A -Selection of the most representative NCCL image cross-sectional reconstruction;B- definition of the guidelines (green lines); C- superimposition of the linear measurement tool; D- final linear measurement (red line) of the distance from the pulp chamber to the internal angle of the lesion

One evaluator, with experience in tomographic appraisal, was previously calibrated regarding tomographic aspects of non-carious cervical lesions, characteristics to be assessed and software tools that should be used during image evaluation. During these evaluations, the software’s curved sling tool was used to acquire cross-sectional reconstructions along the long axis of the tooth. The inclination and definition of these reconstructions were established for each tooth along its long axis. Once the tooth/reconstruction inclination was defined, the most representative cervical lesion image (Figure 2A) was used to measure the remaining dentin thickness (RDT): the distance (mm) between the deepest portion of the lesion (internal angle of lesion) and the pulp chamber. The orientation of the linear measurements was standardized by first using the angle mode tool to generate two perpendicular lines: one line along the long axis of the tooth and another one at a 90-degree angle toward the lesion (Figure 2B). The software’s linear measurement tool was used to determine RDT under the lesion by measuring the distance (along the axis perpendicular to the long axis of the tooth) from the pulp chamber to the internal angle of the lesion (Figure 2C), providing a linear measurement in mm (Figure 2D). Half of the sample was revaluated after one month to evaluate intraobserver reproducibility. Intraobserver agreement was calculated by intraclass correlation coefficients using MedCalc software, version 15.2 (MedCalc Software, Mariakerke, Belgium).

After the assessments previously described, the obtained data were analyzed at the 5% level of significance using Sigma Plot (version 12.0; Systat Software Inc, Chicago, IL, USA). An analysis of the RDT for the tooth type study factor was performed groups were compared by the Kruskal Wallis test. Data from pain level for dentin hypersensitivity, pulpal and the periradicular health in NCCL teeth from G1 and G2 groups were compared by the Mann-Whitney test. The t-test compared the RDT on G1 and G2. Then data from lesion size and RDT were correlated using the Pearson’s test. Spearman correlation to correlate lesion size and pain level of dentin hypersensitivity. were considered statistically significant (P-values of <0.05).

## Results

**Figure 3:**
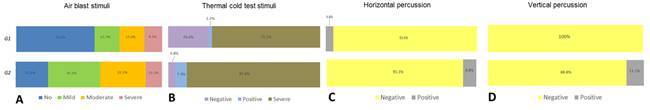
Dental pain response (%) to clinical diagnostic tests according to the group: A- Percentage of dentin hypersensitivity levels according to VAS; B- Findings to pulp health test (cold); C- Horizontal percussion response; D- Vertical percussion response.

Regarding the variability of the types of teeth in the study, the statistical test showed no difference between the tooth types considering the RDT (p=0.902). The results of the hypersensitivity clinical tests for vertical and horizontal percussion, cold sensitivity test, are described in figure 3. The average of the RDT values according to the depth of the lesion are described in table 1.

**Figure 4:**
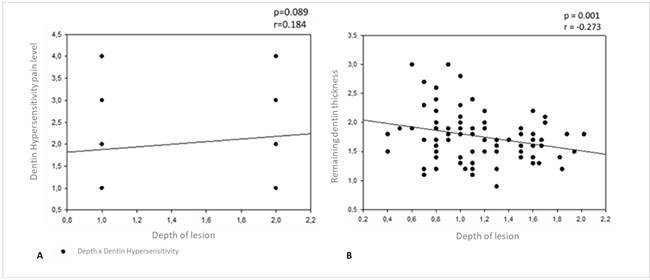
A- Spearman’s correlation between lesion depth and pain level for hypersensitivity; B- Pearson’s correlation depth of the lesions and the remaining dentin thickness.

Depth of lesion did not influence the evaluations of hypersensitivity (p=0.129), cold thermal test (p=0.125), horizontal (p=0.119) or vertical percussion (p=0.317). There was no correlation between lesion depth and pain level for sensitivity (p=0.089/r=0.184) (Figure 4).

The depth of the lesion influenced the RDT (p<0.001), with the G2 (1.627 ± 0.312 mm) group having a lower RDT compared to G1 (1.922 ± 0.459 mm) (Table 1). There was a negative correlation between the depth of the lesions and the RDT (p=0.011/r=-0.273) (Figure 4). So the first hypothesis was accepted. The second was rejected since the depth of the lesion influenced the remaining dentin.

**Table 1:** Average of the remaining dentin thickness (mm) values according to the depth of non-carious cervical lesion.

Groups	G1 (≤ 1.0)	G2 (≥1.1 - ≤ 2.0)
Remaining Dentin Thickness (RDT)	1.922 ± 0.459	1.627 ± 0.312
p value	<0.001	

T- test (p<0.05).

## Discussion

Exposed dentin in the oral cavity, as in teeth with non-carious cervical lesions, subjects dentin tubules to chemical, mechanical and thermal stimuli ^20^. These exposed tubules filled with fluid may change after exposure to stimuli and activate intra-dental nerve fibers, via a mechanoreceptor response, causing pain ^21^. Dentin exposure irritates dental pulp, which causes pulp nerves to release neuropeptides that in turn produce local inflammation ^22^, a mechanism that can explain why most of teeth in the present study, regardless of group, presented an exacerbated response for pain on the pulp health test (more than 70%).

The response of dental pulp to stimuli increases not only because dentin tubules are exposed but also because inflammatory mediators are present ^23^. Teeth that do not have hypersensitivity present lower levels of inflammation, whereas teeth with hypersensitivity have a reduction in the activation threshold and an increase in the innervated area of the dentinal tubules ^24^. However, the present study did not show a relationship between the degree of pain detected in the pulp sensitivity test and the level of dentin hypersensitivity reported in hypersensitivity test. The results of the present study suggest that inflammation was limited to the dental pulp tissue since pain was not elicited by the percussion tests in either of the groups (G1 and G2). Worn dentition is rarely sensitive if occurring over many years, whereas rapid wear in a young adult is often sensitive ^25^. This may explain the level of dentinal hypersensitivity both groups had; mostly absent or mild pain level. Despite of the patients’ age ranged from 25 to 50 years old, in this research, the patient’s age means was 48 year old and most patients were above 48.

Non-carious cervical lesions depth did not influence the degree of dental pulp sensitivity in the present study. This is probably because pulp tissue inflammation was similar given that both groups suffered from exposed dentin, continuous infiltration and bacterial penetration through unsealed dentin tubules ^20^. After external stimuli the tooth presented dentin sclerosis formation ^25^. Teeth without painful levels exhibit more occluded tubules ^26^. The result is formation of sclerotic dentine or of dead tracts and underlying ‘‘secondary’’ reparative dentine formation ^27^. It could have been expected that teeth with greater loss of structure could also present greater deposition of reparative dentine, with a consequent reduction in the volume of the pulp chamber. However, in our study, the thickness of the remaining dentin was inversely correlated with the depth of the lesion. As previously described, sclerotic dentine consists of many tubules that are completely filled with a material similar to the one of peritubular dentin, but that the capacity and speed of the repair processes depend on factors such as age, which can hinder or increase the dentin deposition capacity ^27^ ^,^ ^28^. In the G2 group, a smaller remaining dentin thickness was observed, indicating that the repair capacity of the pulp in terms of dentin thickness is smaller than the loss of structure that occurs in teeth with NCCL. Although deeper non-carious cervical lesions are closer to the pulp, dentin hypersensitivity levels may also have been influenced by the quality of the dentin formed ^25^, leading to a similar pain level presented by teeth with shallower lesions

 The significant differences observed in the present study are an isolated consequence of the depth of the NCCL and is not a consequence of the anatomical variation among different teeth, as presents in results section, with this analysis, it is possible to confirm that data of the study are reliable. Limitations were present in present study, first the wide age range of patients and even individual characteristics that could influence the different levels of pain sensation (subjective) and quality of dentin deposition. Furthermore, it was not possible to standardize or determine the time of non-carious cervical lesions formation or the supporting factors of cervical lesions in each individual. However, the literature shows that the process of dental aging is not just associated to age. This process is affected by multiple factors, such as lifestyle, environment and genetics. These changes can be considered as diseases, such like(as) non-carious cervical lesions ^29^. Thus, we understand that although the age difference, teeth with non-carious cervical lesions are in an aging process independent of the individual’s age. Other limitation were the etiological factors, such as, age, sex, oral hygiene behavior, type of saliva, diet, brushing force, status of periodontium, number of teeth, occlusion, occlusion contact area, occlusal corrosion and attrition, severity of gastroesophageal diseases ^4^ ^,^ ^30^. Was not possible to control all these etiological factors due to the particularity of each patient, to minimize patient factor we insert in our study the largest number of teeth in a single patient and insert some inclusion criteria.

For future studies, there is a need to clinically investigate cervical lesions with greater depths, standardized ages and associated individual etiological factors. This study shows that teeth with a depth of non-cervical lesions up to 2mm do not show differences in pulp or periradicular response in clinical diagnostic tests. Furthermore, cone beam computed tomography assessment indicated that the RDT is related to the depth of the lesion. A better understanding of non-carious cervical lesions signs and symptoms will lead to better diagnosis and treatment planning, improving patients' quality of life and professional clinical practice.

In relation to the tomographic appraisal, although cone beam computed tomography allows examination of entire structures and provides precise dimensions, shapes and locations, its high cost and radiation exposure are limiting factors for its indication for this specific use. In this sense, cervical lesions are often observed in tomographic images acquired for different or supplementary purposes that generally use 0.2mm voxel sizes, as well as the present study. High-resolution cone beam computed tomography scans, using smaller voxel sizes, could provide better images for such small lesions, but it would not represent the most common scenario in dental offices. Further studies comparing the influence of this parameter in lesions examination should be performed.

For future studies there is a need to clinically study non-carious cervical lesions with greater depths, with standardization of age and associated individual etiological factors. This study shows that teeth with non-carious cervical lesions depth up to 2mm did not show differences in pulp or periradicular response in clinical diagnostic tests. Furthermore, the tomography assessment of the remaining dentin thickness is related to the depth of the lesion. A better understanding of the signs and symptoms of non-carious cervical lesions will lead to better diagnosis and treatment planning, improving patients' quality of life and professional clinical practice.

## Conclusion

Within the limitations of this observational study, it was concluded that the depth of non-carious cervical lesions between 0.1 and 2mm neither affect clinical response in dentin hypersensitivity intensity nor the pulp, and periradicular health condition. However, lesions with ≤1.0mm in-depth showed more remaining dentin thickness in the tomographic findings. 
